# The Anti-apoptotic Murine Cytomegalovirus Protein vMIA-m38.5 Induces Mast Cell Degranulation

**DOI:** 10.3389/fcimb.2020.00439

**Published:** 2020-08-25

**Authors:** Julia K. Schmiedeke, Ann-Kathrin Hartmann, Teresa Ruckenbrod, Michael Stassen, Matthias J. Reddehase, Niels A. Lemmermann

**Affiliations:** ^1^Institute for Virology and Research Center for Immunotherapy (FZI) at the University Medical Center of the Johannes Gutenberg-University of Mainz, Mainz, Germany; ^2^Institute for Immunology and Research Center for Immunotherapy (FZI) at the University Medical Center of the Johannes Gutenberg-University of Mainz, Mainz, Germany

**Keywords:** bone marrow-derived mast cells (BMMC), degranulation, mast cells, mast cell-specific Cre recombination, murine cytomegalovirus, gene *m38.5*, peritoneal exudate-derived mast cells (PEMC), vMIA

## Abstract

Mast cells (MC) represent “inbetweeners” of the immune system in that they are part of innate immunity by acting as first-line sentinels for environmental antigens but also provide a link to adaptive immunity by secretion of chemokines that recruit CD8 T cells to organ sites of infection. An interrelationship between MC and cytomegalovirus (CMV) has been a blank area in science until recently when the murine model revealed a role for MC in the resolution of pulmonary infection by murine CMV (mCMV). As to the mechanism, MC were identified as a target cell type of mCMV. Infected MC degranulate and synthesize the CC-chemokine ligand-5 (CCL-5), which is released to attract protective virus-specific CD8 T cells to infected host tissue for confining and eventually resolving the productive, cytopathogenic infection. In a step forward in our understanding of how mCMV infection of MC triggers their degranulation, we document here a critical role for the mCMV *m38.5* gene product, a mitochondria-localized inhibitor of apoptosis (vMIA). We show an involvement of mCMV vMIA-m38.5 in MC degranulation by two reciprocal approaches: first, by enhanced degranulation after *m38.5* gene transfection of bone marrow-derived cell culture-grown MC (BMMC) and, second, by reduced degranulation of MC in peritoneal exudate cell populations infected *ex corpore* or *in corpore* with mutant virus mCMV-Δ*m38.5*. These studies thus reveal a so far unknown function of mCMV vMIA-m38.5 and offer a previously unconsidered but biologically relevant cell system for further analyzing functional analogies between vMIAs of different CMV species.

## Introduction

Human cytomegalovirus (hCMV) is a clinically relevant pathogen in transplantation medicine. Transient immunodeficiency after ablative therapy of hematopoietic malignancies followed by hematopoietic cell transplantation (HCT) opens a “window of risk” from reactivation of latent virus (Reddehase and Lemmermann, 2019) and disseminated cytopathogenic organ infection in the recipients. Similarly, immunosuppressive treatment for avoiding graft rejection by a host-vs.-graft (HvG) response can lead to graft loss caused by reactivated hCMV infection in MHC or minor-histocompatibility loci mismatched solid-organ transplantation (SOT) (for clinical overviews, see Ho, 2008; Boppana and Britt, 2013; Seo and Boeckh, 2013).

As experimental approaches to study *in vivo* mechanisms are not or only to a very limited extent feasible in humans, definitely excluding infection with virus recombinants that carry targeted mutations, mouse models based on murine cytomegalovirus (mCMV) have been developed to mimic clinical correlates as close as possible. These models have helped to understand basic mechanisms of viral pathogenesis and immune control (Krmpotic et al., 2003; Reddehase, 2016; Reddehase and Lemmermann, 2018). As a common denominator, murine models of syngeneic HCT (Holtappels et al., 1998; Podlech et al., 1998, 2000; Reddehase, 2016), MHC-mismatched allogeneic HCT (Holtappels et al., 2020) and MHC-matched but minor histocompatibility antigen mismatched allogeneic HCT (Gezinir et al., 2020) have revealed a critical role for CD8 T-cell reconstitution in preventing cytopathogenic mCMV spread and lethal viral histopathology.

Mast cells (MC) are cells of the innate immune system usually known as effectors in allergic diseases (Galli and Tsai, 2012). They also have been implicated in the pathophysiology of gastrointestinal disorders, many types of malignancies, and cardiovascular diseases (reviewed by Krystel-Whittemore et al., 2016). Physiological functions include the regulation of vasodilation, vascular homeostasis, innate and adaptive immune responses, and angiogenesis (reviewed by Krystel-Whittemore et al., 2016). The metaphor of the MC as being a “loaded gun” is based on potent pro-inflammatory mediators that are stored in secretory granules ready for an almost instantaneous release upon MC stimulation (Rodewald and Feyerabend, 2012). In addition, MC activation by extracellular signals, including the interaction with viruses, can induce chemokine synthesis and secretion such as of RANTES/CCL5 (King et al., 2002; Venkatesha et al., 2005; McAlpine et al., 2012). Besides MC degranulation induced by antigen binding to the canonical MC receptor complex IgεRI-IgE involved in anaphylactic responses, ligation of a broad receptor repertoire, which includes pattern recognition receptors such as the toll-like receptors (TLR) TLR3 and TLR9, can activate MC (Sandig and Bulfone-Paus, 2012). Ca^2+^ signaling is essential for MC degranulation. This involves regulation of Ca^2+^ fluxes through Ca^2+^ pumps and ion exchangers in mitochondria, endoplasmic reticulum, and plasma membrane to sustain elevated levels of cytosolic Ca^2+^, which is an obligatory signal for MC activation (for reviews, see Ma and Beaven, 2011; Wernersson and Pejler, 2014).

MC were implicated to be involved in the host response to CMV infection when the murine model revealed a novel crosstalk axis between MC and the adaptive immune defense against mCMV (Ebert et al., 2014). Studying the pathomechanism of interstitial pneumonia, which is a most relevant clinical manifestation of CMV infection after HCT, protective antiviral CD8 T cells were found to be less efficiently recruited to the lungs of infected MC-deficient C57BL/6-Kit^W−sh/W−sh^ “sash” mutant mice compared to MC-sufficient C57BL/6 WT mice. This was reflected by impaired confinement of infected cells within nodular inflammatory foci (NIF) formed by tissue-infiltrating CD8 T cells and, as a consequence, by enhanced viral spread and histopathology. Efficient lung recruitment of antiviral CD8 T cells in MC-sufficient as well as in MC-reconstituted “sash” mutant mice correlated with degranulation of infected CD117^+^FcεRI^+^ MC and a serum wave of MC-derived chemokine CCL-5 (Ebert et al., 2014). Continuing studies revealed an almost instant MC degranulation that indirectly depended on TLR3/TRIF signaling, and a delayed MC degranulation triggered by direct infection of MC, not involving TLR3/TRIF signaling (Becker et al., 2015). As this delayed MC degranulation proved to account for the biological function in antiviral control in the lungs (Lemmermann and Reddehase, 2017), this mechanism of MC degranulation moved into the focus of interest.

Here we demonstrate that degranulation of mCMV-infected MC depends on expression of the mitochondria-localized inhibitor of apoptosis vMIA-m38.5, the product of gene *m38.5*, which is the ortholog of hCMV gene *UL37.1* that encodes the hCMV vMIA-UL37.1 (Goldmacher et al., 1999; McCormick et al., 2005; Arnoult et al., 2008; Jurak et al., 2008; Norris and Youle, 2008; reviewed by Handke et al., 2012).

## Materials and Methods

### Viruses, Mice, and Cells

High titer virus stocks of bacterial artificial chromosome (BAC)-derived mCMV-*egfp* (Angulo et al., 2000), mCMV-flox-*egfp* and mCMV-rec-*egfp* (Sacher et al., 2008) were prepared from infected murine embryonic fibroblasts (MEF) according to standard protocol (Podlech et al., 2002). C57BL/6 mice were purchased from Janvier Labs. Mcpt5*-cre* transgenic mice (Scholten et al., 2008) were bred in-house. All mice were housed under specified pathogen-free conditions in the translational animal research center (TARC) of the University Medical Center of the Johannes Gutenberg-University Mainz, Germany. MEF were prepared from C57BL/6 mice by a standard protocol (Podlech et al., 2002).

### Generation of BMMC

Bone marrow (BM)-derived mast cells (BMMC) from C57BL/6 and Mcpt5*-cre* mice were generated by using standard procedures (Stassen et al., 2006). In brief, BM cells were isolated and subsequently cultivated in Iscove's modified Dulbecco's medium (IMDM), supplemented with 10% FCS, 2 mM L-glutamine, 1 mM sodium pyruvate, 100 U/ml penicillin, 100 μg/ml streptomycin, 20 U/ml murine IL-3, 200 ng/ml stem cell factor (SCF; c-kit ligand), and 50 U/ml murine IL-4. Non-adherent cells were transferred to fresh culture plates every week to remove adherent macrophages and fibroblasts. After 4–5 weeks of culture, the resulting BMMC population had reached a purity of more than 95%.

### Generation of Recombinant Virus mCMV-Δ*m38.5*-*egfp*

mCMV-Δ*m38.5*-*egfp* was generated by partial deletion of ORF *m38.5* between nucleotides 51,958 and 52,368 (Rawlinson et al., 1996, GenBank accession number NC_004065) through *en-passant* mutagenesis of the BAC plasmid pSM3fr-rev (Angulo et al., 2000) as described (Tischer et al., 2010). Briefly, a *kanR* cassette flanked by homologous viral sequences was amplified from plasmid pori6K-RIT (Hammer et al., 2018) using the oligonucleotides Δ*m38.5*_for GCG AAC ATC CTC TCG GTG TTC GGC ACG ATT GTC GTT GTC GAC ACC GCC ATC GCT GTC CCC TCC ACA ACT ACA ACC GAC GCA TCG TGG CCG GAT CTC and Δ*m38.5*_ rev TAA AAG TTG CGC GGA CGG TGC CGC GGG TTG TAG TTG TGG AGG GGA CAG CGC GAT TGT CGT TGT CGA CAC CGC CAT GTG ACC ACG TCG TGG AAT GC. The resulting product was transformed into GS1783 bacteria carrying pSM3fr-rev. After Red recombination, arabinose-induced *I-Sce*I expression, and a second round of Red recombination, BAC pΔ*m38.5*-*egfp* was purified, and successful mutagenesis was confirmed by sequencing (Eurofins Genomics). After BAC DNA purification, the recombinant mCMV was reconstituted by transfection of the DNA into MEF and propagated for six passages until residual BAC sequences were lost as verified by PCR (Lemmermann et al., 2010).

### Procedures of Infection

(i) Infection of BMMC. BMMC were centrifugally infected with 0.2 plaque-forming-units (PFU) of mCMV per cell, corresponding to a multiplicity of infection (MOI) of 4 (Podlech et al., 2002, and references therein), stimulated with 1 μM ionomycin in supplemented IMDM (see above), and inspected under the Axiovert 200 M fluorescence microscope (Carl Zeiss). (ii) Infection of peritoneal exudate cells (PEC). PEC were isolated from the peritoneal cavity of C57BL/6 mice by standard protocol (Ray and Dittel, 2010). 1 × 10^6^ PEC were seeded in IMDM medium supplemented with 5% FCS, 100 U/ml penicillin, and 100 μg/ml streptomycin and centrifugally infected with 0.2 PFU per cell (MOI 4) of mCMV directly after seeding. (iii) Infection of mice. Intraperitoneal infection of C57BL/6 mice was performed with 5 × 10^5^ PFU of mCMV diluted in 300 μl PBS. Infected PEC were isolated 18 h later for analysis.

### Determination of Productive mCMV Infection in BMMC

C57BL/6 and Mcpt5*-cre* BMMC were infected with mCMV-flox-*egfp* or mCMV-rec-*egfp*. Five days later, cell culture supernatants were harvested and released infectious virus was quantitated on permissive C57BL/6 MEF by standard plaque assay under conditions of centrifugal enhancement. Total plaque formation and eGFP^+^ (green) plaques were counted 3 days later under the Axiovert 200M fluorescence microscope.

### Construction of m38.5 Expression Plasmid

To generate m38.5 expression plasmid, PCR was performed using mCMV MW97.01 (Wagner et al., 1999) genomic DNA with primers pIRES-*m38.5*-XhoI-for ATA TCT CGA GAT GGA GAG TGT GCG CC and pIRES-*m38.5*-EcoRI-rev GCG CGG AAT TCC TAG AAT GTG TAA TCT C, amplifying the full length ORF *m38.5*. The PCR product was subcloned within the *Xho*I and *EcoR*I restriction sites into vector pIRES2-*egfp* (Takara Bio). Successful generation of pIRES2-*m38.5-egfp* was confirmed by sequencing (Eurofins Genomics).

### Transfection of BMMC

BMMC (1 × 10^6^ cells in 0.2 ml serum-free IMDM) were transfected with 7 μg plasmid DNA by electroporation in 0.2-cm cuvettes at room temperature using a Bio-Rad Gene Pulser (Richmond, CA) set at 290 V, 200 Ω, and 600 μF. Cells were allowed to recover for 4 h in IMDM supplemented with 5% FCS and antibiotics, harvested, washed with PBS containing 1% BSA and 2 mM EDTA, and analyzed by flow cytometry as outlined below.

### Measurement of MC Degranulation

BMMC were harvested 4 h after transfection. Cell surface staining was performed with the following antibodies for cytofluorometric analysis: BV421-conjugated anti-CD117 (clone ACK2, eBioscience), PE-Cy7-conjugated anti-FcεRI (clone MAR-1; BioLegend, San Diego, CA, USA), and eFluor660-conjugated anti-CD107a (clone 1D4B; eBioscience). PEC were isolated and stained accordingly *ex vivo* after either *in corpore* or *ex corpore* infection. Optionally, to facilitate gating for peritoneal exudate-derived MC (PEMC), PEC were additionally stained with APC-conjugated anti-Ly-6A/E (Sca-1) (clone D7; BioLegend). Derivation of PEC and PEMC from hematopoietic lineages was verified by staining with BV510-conjugated anti-panCD45 (clone 30-F11, BioLegend). PEC preparations contain almost no cells other than leukocytes. Doublets were excluded by gating on singlets in the sideward scatter height (SSC-H) vs. area (SSC-A) plot prior to setting a “live cell gate” based on scatter properties SSC-A and forward scatter area (FSC-A). Gating for cells expressing eGFP and MC cell surface markers was done as established in our own previous work, in which an isotype control for degranulation marker CD107a was used to exclude unspecific antibody binding to infected MC (Becker et al., 2015). Cytofluorometric analyses of cell surface staining and intracellular eGFP fluorescence were performed with BD FACSymphony (BD Biosciences) and BD FACSDiva V6.1.3 using FlowJo V.10.6.2 software (BD Biosciences).

### Statistical Analysis

To evaluate statistical significance of differences between two independent sets of data, Student's *t*-test (two-sided, unpaired) was used with Welch's correction to account for unequal variance. Differences were considered as being statistically significant at levels of *P* < 0.05 (*), < 0.01 (**), and < 0.001 (***). Calculations were performed with Graph Pad Prism 8.4.1 (Graph Pad Software).

## Results

### MC-specific Cre-recombination Proves Productive Infection of BMMC

Previous work with eGFP-tagged virus has shown that mCMV can infect peritoneal exudate-derived CD117^+^FcεRI^+^ MC (PEMC) *in corpore* and can trigger their degranulation (Ebert et al., 2014). Recombination of mCMV-flox-*egfp* (Sacher et al., 2008) in *cre*-transgenic B6-Mcpt5-*cre* mice, which express Cre recombinase selectively in MC, generated infectious mCMV-rec-*egfp* (Becker et al., 2015) that also spread to other cell types such as hepatocytes and endothelial cells in the liver (Podlech et al., 2015). In contrast, BMMC were found to be not permissive for infection, not even expressing the immediate-early (IE) phase protein IE1 (Ebert et al., 2014). This discrepancy between PEMC and BMMC was explained by different stages of MC maturation (Matsushima et al., 2004). So, a cell-culture system for infection of MC was missing so far.

**Figure 1:**
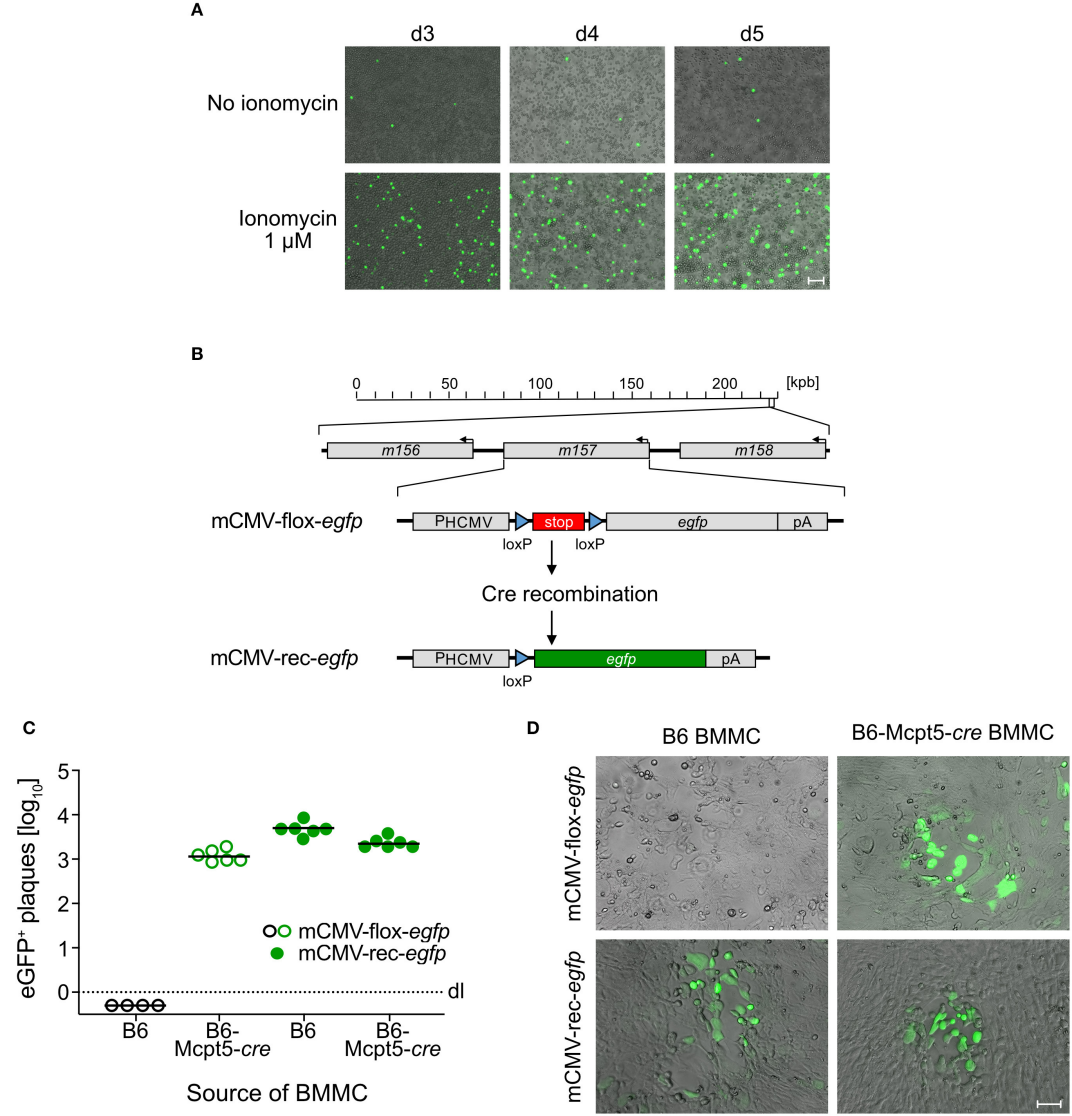
mCMV productively infects BMMC. **(A)** C57BL/6-derived BMMC were centrifugally infected with mCMV-*egfp* at an MOI of 4 and stimulated with 1 μM ionomycin for the cultivation period of 3–5 days. Representative images show infected eGFP^+^ (green) BMMC, indicating virus spread in the cultures over time. Bar marker: 50 μm. **(B)** Scheme explaining the principle of Cre-mediated recombination generating mCMV-rec-*egfp* that expresses green fluorescent eGFP [modified from Becker et al. (2015)]. **(C)** Quantitation of eGFP^+^ plaques in C57BL/6 MEF indicator monolayers on day 3 after infection with supernatants from ionomycin-conditioned C57BL/6 (B6) and Cre-transgenic Mcpt5*-cre* (B6-Mcpt5-*cre*) BMMC infected at an MOI of 4 with either mCMV-flox-*egfp* (open circles) or mCMV-rec-*egfp* (filled circles) and propagated for 5 days. Symbols represent plaque counts for independent BMMC cultures. Median values are indicated. Dotted line: *dl*, detection limit. **(D)** Representative eGFP^+^ plaques in MEF monolayers on day 3 after infection with supernatants from the BMMC cultures. Bar marker: 50 μm.

Interestingly, pre-treatment of BMMC with the Ca^2+^ ionophore ionomycin, which rapidly raises the intracellular level of Ca^2+^, renders MC permissive to mCMV infection (Figure 1A). This gives a first hint to a critical role of Ca^2+^ levels in the infection of MC and suggests that the previously observed difference between PEMC and BMMC (see above) was based on differences in Ca^2+^ mobilization. Although BMMC cultures reach high MC purity, some contamination by macrophages, which are permissive to mCMV infection, cannot be excluded. To verify that productively infected cells in BMMC cultures are MC, we again used the strategy of MC-specific Cre recombination to remove the stop cassette in virus mCMV-flox-*egfp* for eGFP expression selectively in MC (Figure 1B). Indeed, whereas pre-recombined mCMV-rec-*egfp* infected BMMC derived from both C57BL/6 (B6) and *cre*-transgenic B6-Mcpt5-*cre* mice produced infectious eGFP^+^ progeny, mCMV-flox-*egfp* generated infectious eGFP^+^ progeny only from B6-Mcpt5-*cre* BMMC (Figures 1C,D). This gives final evidence for productive infection of BMMC.

### Selective Expression of mCMV vMIA-m38.5 Triggers Degranulation of Murine BMMC

**Figure 2:**
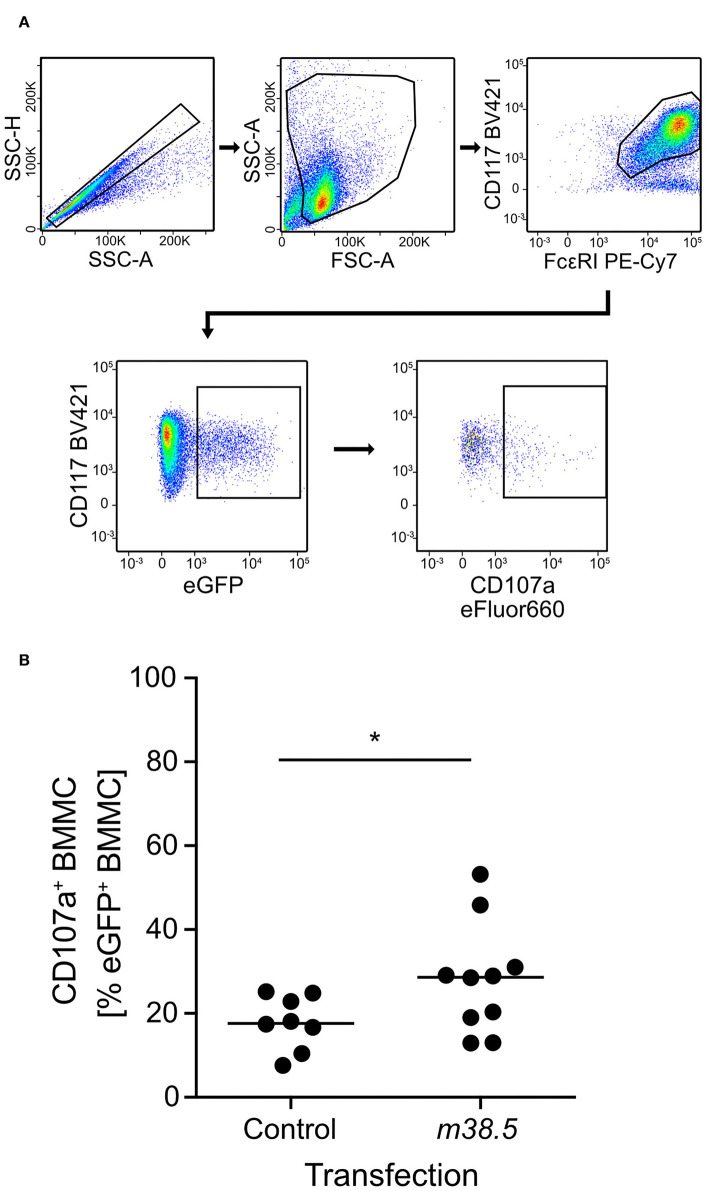
m38.5 induces degranulation of transfected BMMC. **(A)** Gating strategy (representative example from group *m38.5*) to restrict the degranulation analysis to eGFP^+^CD117^+^FcεRI^+^ MC. Degranulation is indicated by cell surface expression of CD107a. 2-parameter dot plots of fluorescence intensities are displayed with biexponential scales. SSC-H, sideward scatter height; SSC-A, sideward scatter area; FSC-A, forward scatter area. **(B)** Quantitation of degranulated CD107a^+^ BMMC at 4 h after *m38.5* transfection (group: *m38.5*) compared to empty vector control (group: Control). Dots represent data from independent transfection cultures compiled from 4 independent experiments. The median values are indicated. *P < 0.05.

As elevated levels of cytosolic Ca^2+^ are known to be decisive for MC degranulation (for reviews, see Ma and Beaven, 2011; Wernersson and Pejler, 2014), and given the experience with the Ca^2+^ ionophore ionomycin in the infection of BMMC (this report), we considered the possibility that a viral protein involved in Ca^2+^ mobilization might be required for the degranulation of mCMV-infected MC. As hCMV vMIA-UL37x1 has been reported to mobilize Ca^2+^ from endoplasmic reticulum stores into the cytosol (Sharon-Friling et al., 2006), we considered mCMV vMIA-m38.5 as a candidate, even though this was thinking laterally because a functional analogy between mCMV and hCMV vMIAs in terms of Ca^2+^ regulation has not yet been established. To test a putative role for mCMV vMIA-m38.5 in MC degranulation, we transfected BMMC with gene *m38.5* expression plasmid pIRES2-*m38.5*-*egfp* and indeed found elevated numbers of cells expressing the degranulation marker CD107a among transfected eGFP^+^CD117^+^FcεRI^+^ BMMC compared to the pIRES2-*egfp* empty vector control expressing only eGFP (Figure 2; for gating controls using pIRES2-egfp, see Figure S1A). This is the first positive evidence for a role of mCMV vMIA-m38.5 in MC degranulation.

### Absence of vMIA-m38.5 Reduces Degranulation of Infected eGFP^+^ PEMC

**Figure 3:**
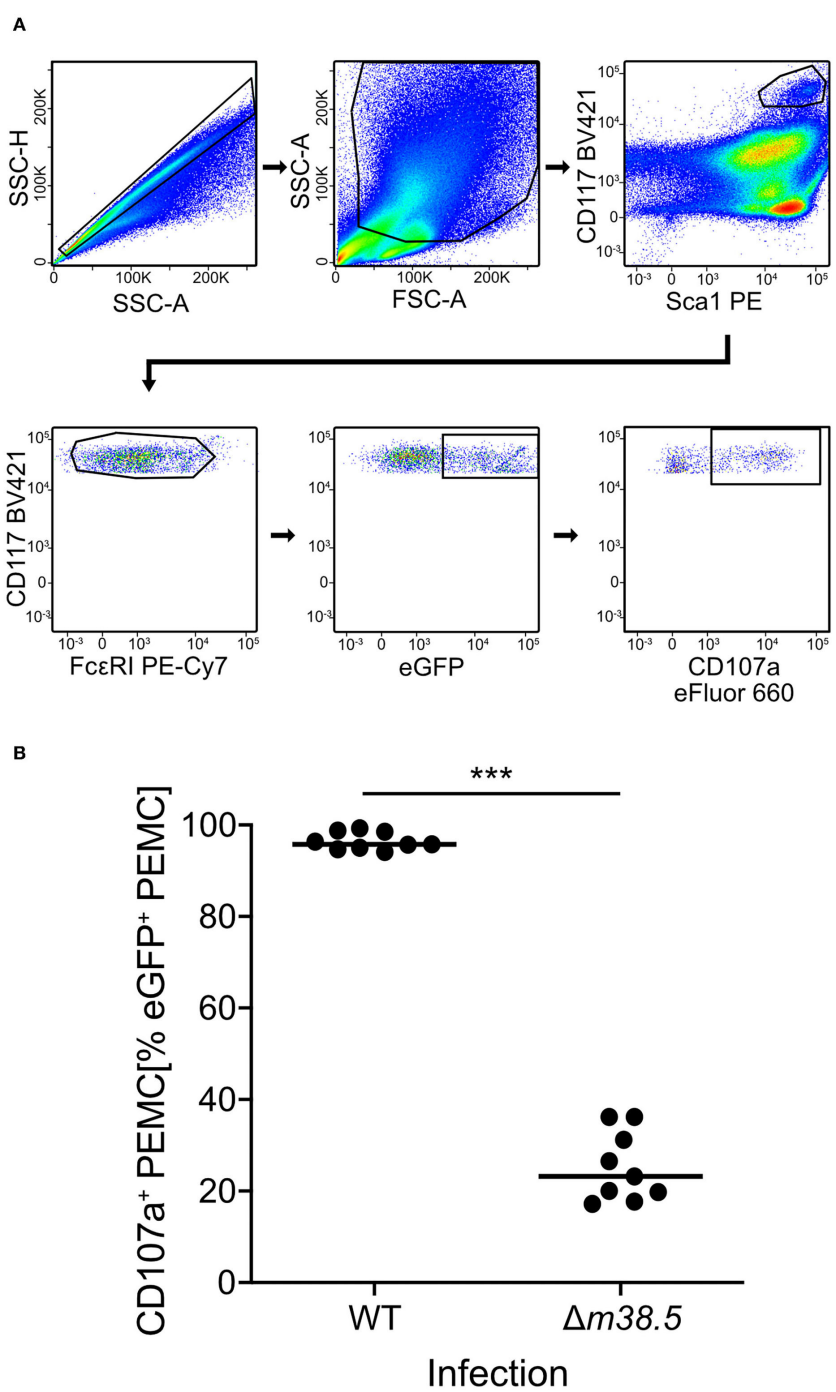
Deletion of gene *m38.5* in virus mCMV-Δ*m38.5-egfp* reduces the degranulation of PEMC infected *ex corpore*. Degranulation was assessed at 18 h after PEC isolation from naïve C57BL/6 mice (pool of 5) and their centrifugal infection with either mCMV-*egfp* (group: WT, wild-type virus) or mCMV-Δ*m38.5*-*egfp* (group Δ*m38.5*) with an MOI of 4. Anti-CD107a antibody was present in the cultures during the infection period. **(A)** Gating strategy (representative example from group Δ*m38.5*) to restrict the degranulation analysis to eGFP^+^CD117^+^FcεRI^+^ PEMC. Degranulation is indicated by cell surface expression of CD107a. 2-parameter dot plots of fluorescence intensities are displayed with biexponential scales. SSC-H, sideward scatter height; SSC-A, sideward scatter area; FSC-A, forward scatter area. **(B)** Quantitation of degranulated CD107a^+^ PEMC. Symbols represent independent infection cultures. The median values are marked. ***P < 0.001.

**Figure 4:**
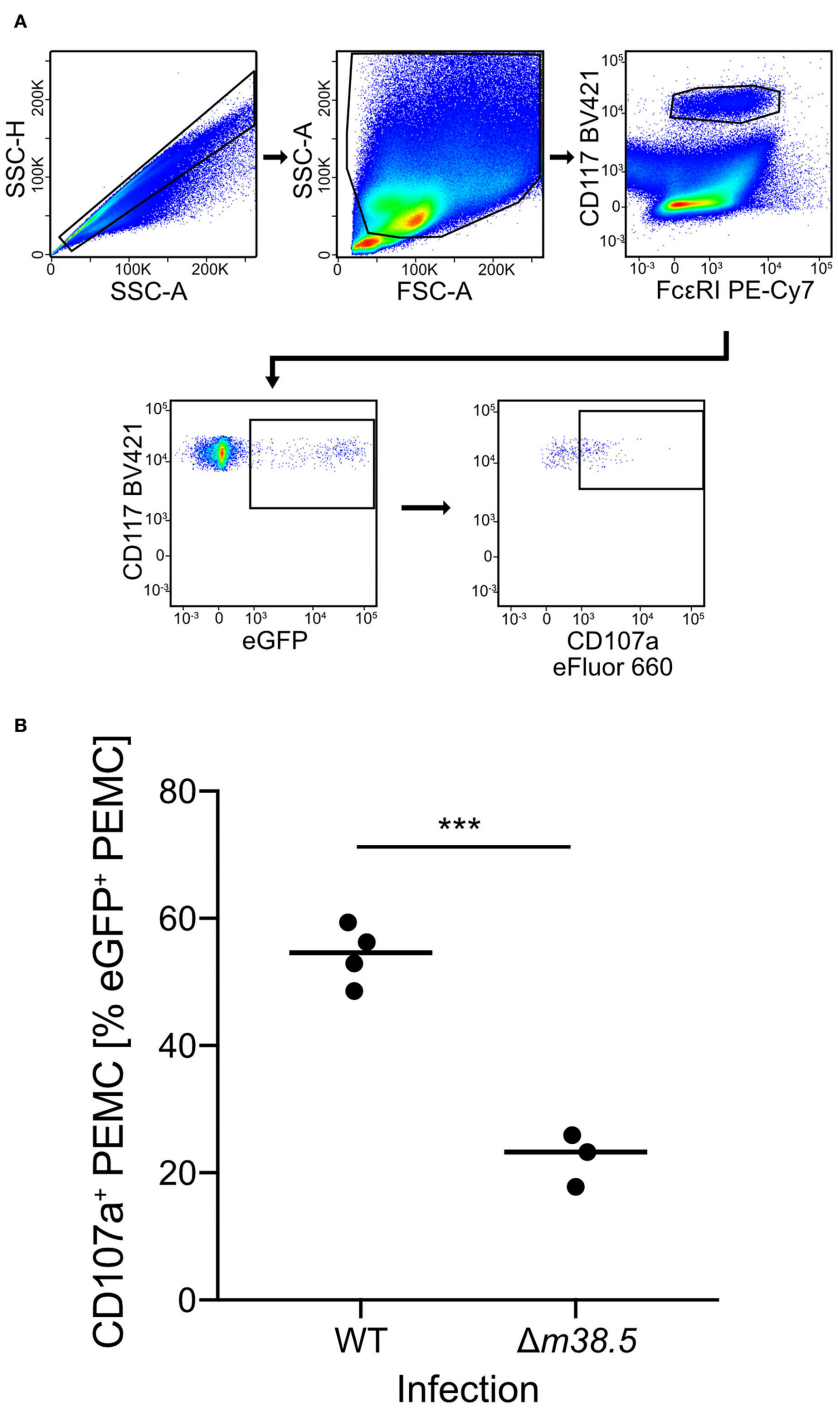
Deletion of gene *m38.5* in virus mCMV-Δ*m38.5-egfp* reduces the degranulation of PEMC infected *in corpore*. C57BL/6 mice were infected intraperitoneally with 5 × 10^5^ PFU/mouse of either mCMV-*egfp* (group: WT, wild-type virus) or mCMV-Δ*m38.5*-*egfp* (group Δ*m38.5*). PEC were isolated at 18 h after infection. **(A)** Gating strategy (representative example from group WT) to restrict the degranulation analysis to eGFP^+^CD117^+^FcεRI^+^ PEMC. Degranulation is indicated by cell surface expression of CD107a. 2-parameter dot plots of fluorescence intensities are displayed with biexponential scales. SSC-H, sideward scatter height; SSC-A, sideward scatter area; FSC-A, forward scatter area. **(B)** Quantitation of degranulated CD107a^+^ PEMC. Symbols represent mice analyzed individually. The median values are marked. ***P < 0.001.

As a reciprocal approach for identifying a role of m38.5 expression in MC degranulation, we isolated CD45^+^ PEC (Figure S2) and infected them *ex corpore* with the *m38.5* gene deletion mutant mCMV-Δ*m38.5*-*egfp* or with virus mCMV-*egfp* encoding m38.5. The deletion significantly reduced the numbers of degranulated CD107a^+^ eGFP^+^CD117^+^FcεRI^+^ PEMC (Figure 3; for gating in the mCMV-*egfp* control, see Figure S1B). Note that CD117^+^FcεRI^low^ cells were included in the analysis in order not to miss infected PEMC that have already downregulated FcεRI in response to activation (Rivera and Gilfillan, 2006; Yoshioka et al., 2007). Finally, to account for putative modulating influences of an *in vivo* cytokine/chemokine milieu in the peritoneal cavity, infection was performed *in corpore* and confirmed the importance of m38.5 expression for triggering MC degranulation (Figure 4; for gating in the mCMV-Δ*m38.5*-*egfp* group, see Figure S1C).

## Discussion

We report here (1) the technical progress of having established a cell culture model of MC infection with mCMV and (2) progress in our understanding of how mCMV infection induces MC degranulation. Specifically, the anti-apoptotic mCMV protein vMIA-m38.5 is identified here as being critically involved in the mechanism that leads to degranulation of infected MC. This is shown for BMMC and PEMC by reciprocal approaches of *m38.5* transfection and deletion that gave the complementary evidence of enhanced MC degranulation in presence of m38.5 and reduced MC degranulation in absence of m38.5, respectively.

Interestingly, mCMV vMIA-m38.5 and hCMV vMIA-UL37.1 are partial functional analogs in that they share the property to inhibit Bax-mediated intrinsic apoptosis (reviewed in Handke et al., 2012). As reported by Norris and Youle (2008), mCMV vMIA-m38.5 as well as hCMV vMIA-UL37.1 interact with the respective cellular pro-apoptotic Bcl-2 family member Bax and recruit it to mitochondria, although m38.5 shares negligible sequence homology with UL37.1 and no region corresponding to the vMIA-UL37.1 Bax-binding domain. The functional analogy is, however, not absolute. Specifically, while hCMV encodes UL37.1 as a single anti-apoptotic viral Bcl-2 (vBcl-2) capable of inhibiting both pro-apoptotic Bcl-2 family members Bax and Bak (Karbowski et al., 2006; Norris and Youle, 2008), mCMV protein m38.5 interacts with Bax only, whereas mCMV protein m41.1 inhibits Bak-mediated apoptosis by functioning as a viral inhibitor of Bak oligomerization, vIBO (Cam et al., 2010).

As Bax and Bak fulfill largely redundant functions in mediating apoptosis, apoptosis of mCMV-infected cells can only be suppressed if Bax and Bak are simultaneously antagonized by m38.5 and m41.1, respectively. Accordingly, an mCMV *m38.5* gene deletion virus has a Bax-mediated apoptosis phenotype, although Bak-mediated cell death remains inhibited *via* m41.1 (reviewed in Handke et al., 2012). The MC degranulation-loss phenotype of virus mCMV-Δ*m38.5*-*egfp* (this report) in presence of m41.1 indicates that interaction between m38.5 and Bax is important for the induction of MC degranulation in mCMV-infected MC. It is open to question if inhibition of intrinsic apoptosis in general is the key to the mechanism. The complementary experiment of selectively allowing Bak-mediated apoptosis by deletion of *m41.1* is still pending.

As far as we are aware of, a functional analogy between hCMV vMIA-UL37.1 and mCMV vMIA-m38.5 or vIBO-m41.1 with respect to regulating the cytosolic Ca^2+^ level has not yet been investigated. Thus, the answer to the question if prevention of apoptosis as such or rather the mobilization of Ca^2+^ is decisive for MC degranulation to occur is still pending, although the established role of cytosolic Ca^2+^ in MC degranulation in systems other than MC infection (Ma and Beaven, 2011; Wernersson and Pejler, 2014), leads us to favor this idea. Thus, continuing experiments are needed to address the open questions (1) if mCMV vMIA-m38.5 elevates the cytosolic Ca^2+^ level as hCMV vMIA-UL37.1 does (Sharon-Friling et al., 2006), and (2) if hCMV vMIA-UL37.1 is required for MC degranulation as it is the case for mCMV vMIA-m38.5 (this report).

All in all, our work provides a previously unconsidered but biologically relevant cell system for further analyzing the functions of CMV vMIAs.

## Data Availability Statement

All datasets presented in this study are included in the article/Supplementary Material.

## Ethics Statement

The animal study was reviewed and approved by the ethics committee of the Landesuntersuchungsamt Rheinland-Pfalz, permission number 177-07/G 19-1-088.

## Author Contributions

NL is responsible for conception, design of the study, analysis, and interpretation of the data. MS and MR are responsible for conception, design of the study, and interpretation of the data. JS and A-KH conducted the work and analyzed the data. TR conducted the work. MR wrote the first draft of the manuscript. JS and NL wrote sections of the manuscript. All authors contributed to manuscript revision, read, and approved the submitted version.

## Conflict of Interest

The authors declare that the research was conducted in the absence of any commercial or financial relationships that could be construed as a potential conflict of interest.

## Supplementary Material

Figure S1 Gating controls **(A)** Corresponding to Figure 2A. Gating strategy for BMMC transfected with empty vector pIRES-*egfp* not expressing the protein m38.5 under study. **(B)** Corresponding to Figure 3A. Gating strategy for PEMC infected *ex corpore* with wild-type mCMV expressing m38.5. **(C)** Corresponding to Figure 4A. Gating strategy for PEMC infected *in corpore* with virus mCMV-Δ*m38.5*-*egfp* not expressing m38.5. 2-parameter dot plots of fluorescence intensities are displayed with biexponential scales. SSC-H, sideward scatter height. SSC-A, sideward scatter area. FSC-A, forward scatter area.

Figure S2 Phenotyping of uninfected PEC. CD117^+^FcϵRI^+^ PEMC co-express Sca-1 and panCD45. While most PEC express panCD45, only a fraction of PEC express Sca-1. 2-parameter dot plots of fluorescence intensities are displayed with biexponential scales. SSC-H, sideward scatter height. SSC-A, sideward scatter area. FSC-A, forward scatter area.
