# Supplementary figures and images for: The Anti-apoptotic Murine Cytomegalovirus Protein vMIA-m38.5 Induces Mast Cell Degranulation
Source: Front Cell Infect Microbiol. 2020 Aug 25;10:439. doi: 10.3389/fcimb.2020.00439 (PMC7477074; doi:10.3389/fcimb.2020.00439)

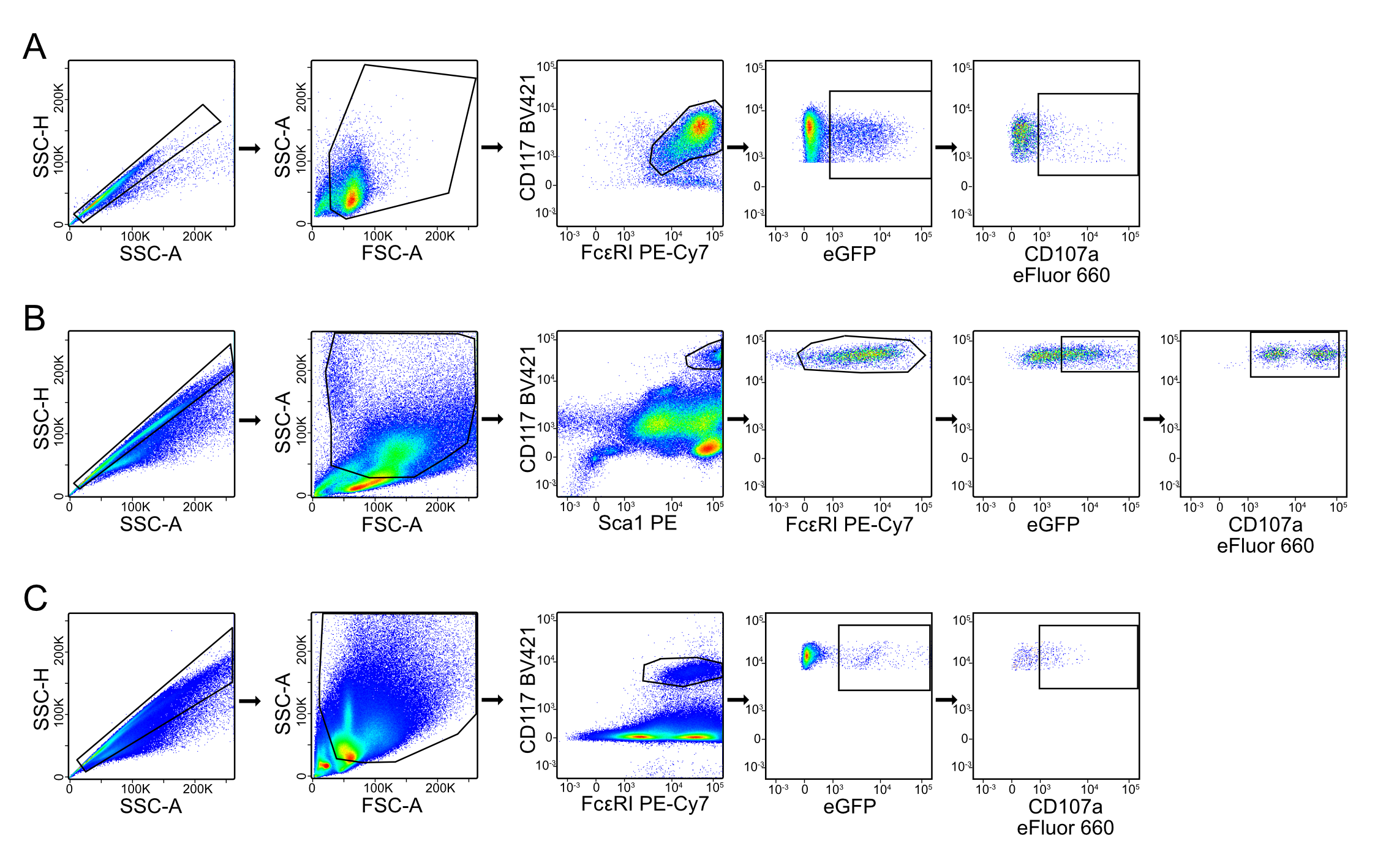

Supplement: Figure S1 — Gating controls (A) Corresponding to Figure 2A. Gating strategy for BMMC transfected with empty vector pIRES-egfp not expressing the protein m38.5 under study. (B) Corresponding to Figure 3A. Gating strategy for PEMC infected ex corpore with wild-type mCMV expressing m38.5. (C) Corresponding to Figure 4A. Gating strategy for PEMC infected in corpore with virus mCMV-Δm38.5-egfp not expressing m38.5. 2-parameter dot plots of fluorescence intensities are displayed with biexponential scales. SSC-H, sideward scatter height. SSC-A, sideward scatter area. FSC-A, forward scatter area. [file Image_1.TIFF]

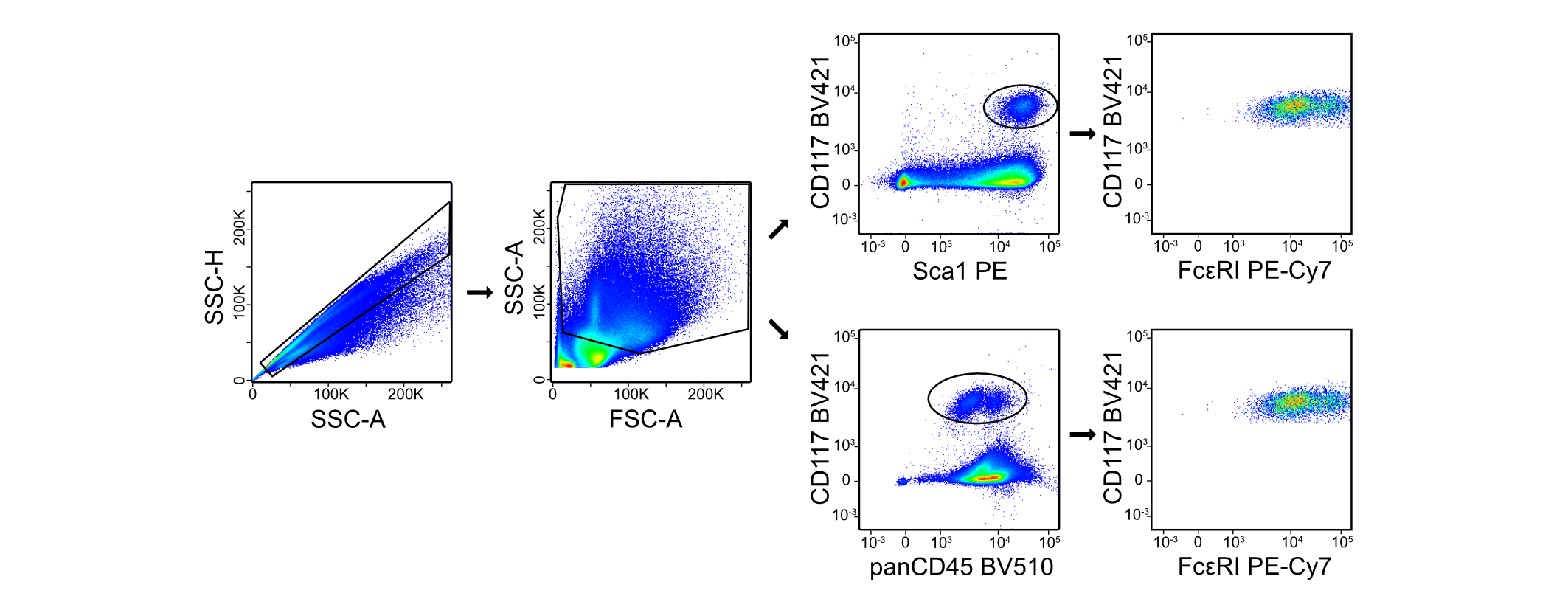

Supplement: Figure S2 — Phenotyping of uninfected PEC. CD117+FcϵRI+ PEMC co-express Sca-1 and panCD45. While most PEC express panCD45, only a fraction of PEC express Sca-1. 2-parameter dot plots of fluorescence intensities are displayed with biexponential scales. SSC-H, sideward scatter height. SSC-A, sideward scatter area. FSC-A, forward scatter area. [file Image_2.TIFF]
